# Prognostic relevance of rib invasion and modification of T description for resected NSCLC patients: A propensity score matching analysis of the SEER database

**DOI:** 10.3389/fonc.2022.1082850

**Published:** 2023-01-04

**Authors:** Yiyong Chen, Juan Zhang, Jing Chen, Zijie Yang, Yun Ding, Wenshu Chen, Tianxing Guo, Lilan Zhao, Xiaojie Pan

**Affiliations:** ^1^ Shengli Clinical Medical College, Fujian Medical University, Fuzhou, Fujian, China; ^2^ Department of Pharmacy, Fujian Children’s Hospital, Fuzhou, Fujian, China; ^3^ Clinical School of Thoracic, Tianjin Medical University, Tianjin, China; ^4^ Department of Thoracic Surgery, Fujian Provincial Hospital, Fuzhou, Fujian, China

**Keywords:** non-small cell lung cancer, rib invasion, pathological T classification, propensity score matching, SEER database

## Abstract

**Introduction:**

The impact of rib invasion on the non-small cell lung cancer (NSCLC) T classifications remains unclear. Our study aims to verify the impact of rib invasion on survival in patients with NSCLC through multicenter data from the Surveillance, Epidemiology, and End Results (SEER) database, and proposed a more appropriate pT for the forthcoming 9^th^ tumor-node-metastasis (TNM) classifications.

**Method:**

The SEER database was used to collect T_2b-4_N_0-2_M_0_ NSCLC cases from the period of 2010-2015 according to the 7^th^ TNM classification system. Subsequently, the T classification was restaged according to the 8^th^ TNM classification system based on the following codes: tumor size and tumor extension. Cases with T1-2 disease and incomplete clinicopathological information were excluded. Finally, 6479 T3 and T4 NSCLC patients were included in the present study and divided into a rib invasion group (n = 131), other pT3 group (n = 3835), and pT4 group (n = 2513). Propensity-score matching (PSM) balanced the known confounders of the prognosis, resulting in two sets (rib invasion group *vs.* other pT3 and pT4 group). Overall survival (OS) and cancer-specific survival (CSS) were investigated using Kaplan-Meier survival curves, and predictive factors of OS and CSS were assessed by Cox regression.

**Result:**

Survival outcomes of the rib invasion group were worse than the other pT3 group (OS: 40.5% *vs*. 46.5%, p = 0.035; CSS: 49.2% *vs*. 55.5%, p = 0.047), but comparable to the pT4 group (OS: 40.5% *vs*. 39.9%, p = 0.876; CSS: 49.2% *vs*. 46.3%, p = 0.659). Similar results were obtained after PSM. Multivariate analyses for all patients revealed that age at diagnosis, gender, N stage, T stage, surgical modalities, and adjuvant therapy had a predictive value for the prognosis.

**Conclusion:**

The rib invasion group had a worse prognosis than the other pT3 groups, but was similar to the pT4 group. Our recommendation is to change the classification of rib invasion to pT4 disease and further validate this in the forthcoming 9^th^ TNM classification.

## 1 Introduction

Lung cancer is the leading cause of cancer death in the world. Non-small cell lung cancer (NSCLC) accounts for 80% of all lung cancers (1). Lung cancer involving adjacent structures are mainly classified as T3 or T4 disease in the 8^th^ tumor-node-metastasis (TNM) classification system which has been in use since January 2017 (2). The T3 tumors are defined as those involving resectable structures (chest wall, pericardium, phrenic nerve or separate tumor nodule(s) in the same lobe), while T4 tumors are those invading organs considered to be unresectable (mediastinum, diaphragm, heart, great vessels, recurrent laryngeal nerve, carina, trachea, esophagus, spine, or tumor nodule(s) in a different ipsilateral lobe).

The incidence of chest wall invasion occurs in 5 to 8% of all surgically treated NSCLC (3). The prognosis of patients with different depths of chest wall involvement is controversial, and the survival difference was reported to be non-significant in a few studies (4–9). Meanwhile, recent studies demonstrated that the depth of chest wall involvement was an independent prognostic risk factor, and the survival rate of patients with deper chest wall invasion was worse than that of patients with chest wall invasion limited to the parietal pleura (10–13). At present, most studies have reported that the depth of chest wall infiltration can affect long-term survival, but only one study specifically investigated the impact of rib invasion on the T classifications (14).

Our study aims to verify the effect of rib invasion on survival in NSCLC patients with a larger sampling of the multicenter data from the Surveillance, Epidemiology, and End Results (SEER) database, and to propose a more appropriate pT in the forthcoming 9^th^ edition of TNM classifications.

## 2 Methods

### 2.1 Study population and data processing

SEER*stat software (version 8.4.0.1) was used to retrieve cases from the SEER database, which is a comprehensive source of demographic information on clinicopathological characteristics and survival of cancer patients in the USA (13). Data were collected only after official permission had been granted (username: 12238-Nov2021) and did not require informed consent from patients. The inclusion criteria were: (I) lung cancer diagnosed pathologically from 2010 to 2015 (Site codes: C34.0, C34.1, C34.2, C34.3, C34.8, and C34.9); (II) pathologically confirmed NSCLC, the common histological types included as follows: adenocarcinoma (ADC; 8140, 8141, 8230, 8244, 8245, 8250–8255, 8260, 8290, 8310, 8320, 8323, 8333, 8410, 8470, 8480, 8481, 8490, 8507, 8550, 8551, 8570, 8571, 8574, 8576), squamous cell carcinoma (SCC; 8052, 8070–8075, 8078, 8083, 8084, 8123), and other non-small cell carcinoma (other NSCLC; 8004, 8012–8014, 8022, 8030–8035, 8046, 8082, 8200, 8240, 8249, 8430, 8560, 8562); (III) lung cancer as the first and only primary cancer diagnosis; (IV) American Joint Committee on Cancer (AJCC) 7^th^ ed. T stage = 2b-4; N stage= 0-2; M stage = 0; and(V) surgery was performed. The T classification was restaged according to the 8^th^ edition AJCC staging classification system based on the following codes: tumor size and tumor extension. In addition, patients with stage T1-2 from the AJCC 8^th^ edition system and incomplete information for race, tumor location, histology, 8^th^ edition AJCC system T stage, tumor size, surgical modalities, and cause-specific death classification were excluded from our analyses.

The following data were also collected: race, age at diagnosis, gender, tumor location, histology, 7^th^ edition AJCC system T and N stage, surgical modalities, chemotherapy, radiotherapy, tumor size, tumor extension, survival months, vital status, cause-specific death classification and cause of death to site recode. Age at diagnosis as a continuous variable was separated into two groups (“< 65 years” and “≥ 65 years”). Tumor size was separated into four groups (“≤ 3 cm”, “> 3 cm”, ≤ 5 cm”, “> 5 cm, ≤ 7 cm”, “> 7 cm”). Treatment with chemotherapy and radiotherapy was separated into four categories (“No/Unknown”, “Radiotherapy”, “Chemotherapy”, “Chemoradiotherapy”). The primary endpoints of this study are cancer-specific survival (CSS) and overall survival (OS). OS was defined as the time between the date of diagnosis to death from any cause or to the last follow-up visit. CSS was defined as the interval from diagnosis to death as a result of lung cancer. Patients who were alive at the last follow‐up or died from other causes were regarded as censored cases in the survival analysis (15).

### 2.2 Statistical analysis

Demographic and tumor characteristics were summarized with descriptive statistics. The Kaplan-Meier method was used to establish the curves of OS and CSS, and the differences were calculated by a log-rank test. Univariate analysis and multivariate analysis were performed with Cox regression analysis. Predictors with p < 0.1 in univariable analysis were incorporated into the multivariate analysis. The results of the univariate analyses and multivariate analyses are presented as hazard ratios (HRs) and 95% confidence intervals (CIs).

Propensity score matching (PSM) was performed to balance the distribution of potentially confounding covariates for patients between groups. The logistic regression model included age at diagnosis, gender, race, tumor size, tumor location, histology, and pathological N stage. We developed a group-based logistic regression model. After calculating propensity scores, the nearest neighbor method was used to identify three other pT3 or pT4 patients for each rib invasion patient. Covariate balance effect was assessed by a standardized difference with a threshold of 0.10. Statistical analyses were performed by using SPSS 26.0 and R version 4.2.0 (http://www.r-project.org/). A bilateral *P* value of less than 0.05 was considered as statistically significant.

## 3 Results

### 3.1 Patient characteristics

**Figure 1:**
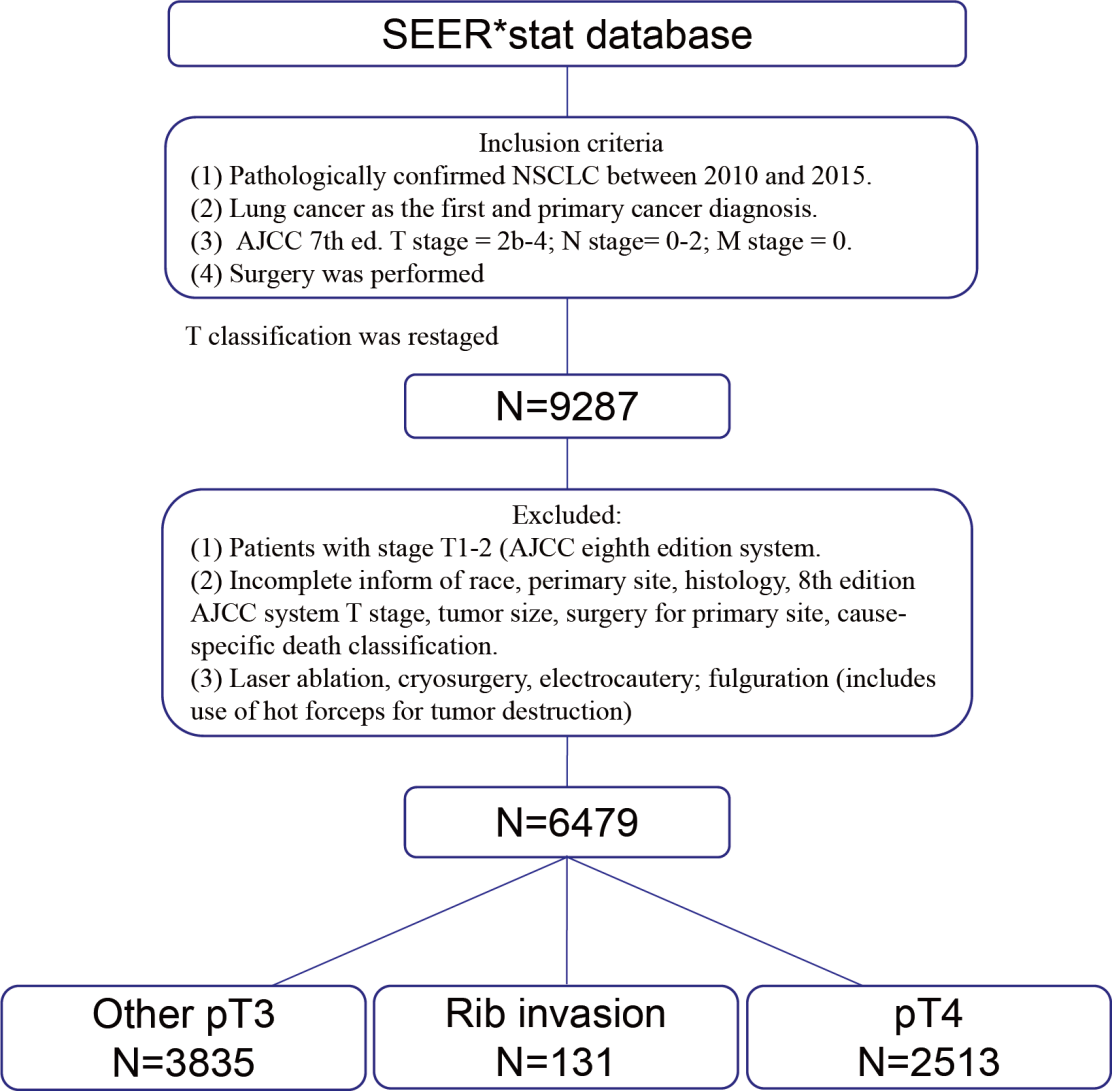
Schematic presentation of case selection. SEER, Surveillance, epidemiology, and end results. NSCLC, Non-small cell lung cancer. AJCC, American Joint Committee on Cancer.

A total of 6,479 cases were included for analyses according to the inclusion criteria and exclusion criteria. A specific screening flowchart is shown in **Figure 1**. The clinicopathological characteristics of all patients, including 3,784 (58.40%) males and 2,695 (41.60%) females, with a median age at diagnosis of 66.40 years are shown in **Table 1**. The entire cohort was divided into three groups: I. Rib invasion group (excluded pT4 disease) (n = 131), II. Other pT3 group (n = 3835), and III. pT4 group (n = 2513). In terms of histology, 3,112 (48.03%) patients were adenocarcinoma, 2,542 (39.24%) patients were squamous cell carcinoma, and 825 (12.73%) patients were other pathology. In addition, 3,917 (60.46%) patients were N0 stage, 1,379 (21.28%) patients were N1 stage and 1,183 (18.26%) patients were N2 stage. Additionally, 467 (7.21%) patients underwent sublobectomy, 5,084 (78.47%) patients underwent lobectomy or bilobectomy, and 928 (14.32%) underwent pneumonectomy. Radiotherapy was performed in 251 (3.87%) cases, chemotherapy was performed in 2,244 (34.63%) cases and chemoradiotherapy was performed in 1,447 (22.33%).

**Table 1:** Clinical characteristic of patients pathological T3-4 NSCLC (N=6479).

Characteristic	Rib (n=131)	T3 (n=3835)	T4 (n=2513)
Age at diagnosis, years			
< 65	54 (41.22)	1414 (36.87)	1082 (43.06)
≥ 65	77 (58.78)	2421 (63.13)	1431 (56.94)
Race, n (%)			
white	112 (85.50)	3230 (84.22)	2069 (82.33)
black	15 (11.45)	325 (8.48)	246 (9.79)
other	4 (3.05)	280 (7.30)	198 (7.88)
Gender, n (%)			
male	80 (61.07)	2183 (56.92)	1521 (60.53)
female	51 (38.93)	1652 (43.08)	992 (39.47)
Tumor location, n (%)			
left	52 (39.69)	1647 (42.95)	1096 (43.61)
right	79 (60.31)	2188 (57.05)	1417 (56.39)
Histology, n (%)			
adenocarcinoma	50 (38.17)	1804 (46.96)	1258 (50.06)
squamous-cell carcinoma	56 (42.75)	1577 (41.12)	909 (36.17)
other	25 (19.08)	454 (11.84)	346 (13.77)
Pathological N stage, n (%)			
N0	108 (82.44)	2424 (63.21)	1385 (55.11)
N1	14 (10.69)	774 (20.18)	591 (23.52)
N2	9 (6.87)	637 (16.61)	537 (21.37)
Surgical modalities, n (%)			
sublobe	12 (9.16)	278 (7.25)	177 (7.04)
lobectomy or bilobectomy	116 (88.55)	3183 (83.00)	1785 (71.03)
pneumonectomy	3 (2.29)	374 (9.75)	551 (21.93)
Pathological size (cm)			
≤ 3	12 (9.16)	419 (10.93)	167 (6.65)
> 3 cm, ≤ 5 cm	53 (40.46)	517 (13.48)	219 (8.71)
> 5 cm, ≤ 7 cm	66 (50.38)	2899 (75.59)	209 (8.32)
> 7 cm			1918(76.32)
Adjuvant therapy, n (%)			
no/unknow	28 (21.37)	1682 (43.86)	827 (32.91)
radiotherapy	9 (6.87)	142 (3.70)	100 (3.98)
chemotherapy	27 (20.61)	1298 (33.85)	919 (36.57)
chemoradiotherapy	67 (51.15)	713 (18.59)	667 (26.54)

Rib, rib invasion group; T3, the other pT3 group (the pT3 patients without rib invasion); T4, pT4 group.

### 3.2. Survival analysis among the unmatched population

**Figure 2:**
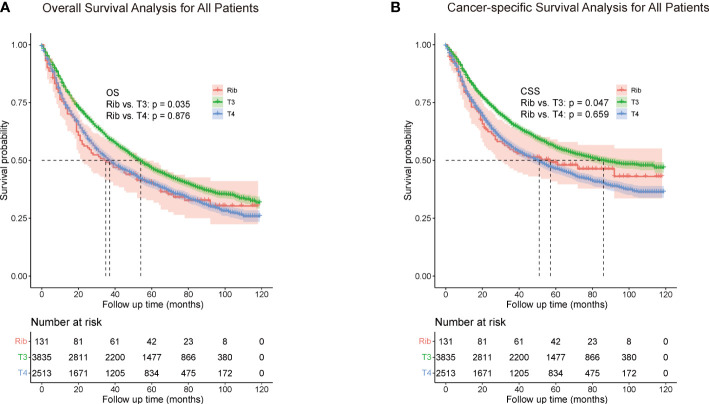
Overall survival **(A)** and cancer-specific survival **(B)** for all patients. The whole cohort was entered into the rib invasion, other pT3, and pT4 groups. Other pT3 referred to the pT3 patients without rib invasion.

Among all patients, the 5-year OS rate of rib invasion group was 40.5% (95% CI, 0.362–0.448), compared with the other pT3 group (46.5%, 95% CI, 0.457 - 0.473; p = 0.035) and pT4 group (39.9%; 95% CI, 0.389 - 0.409; p = 0.876; **Figure 2A**). The 5-year CSS rate of the rib invasion group was 49.2% (95% CI, 0.446–0.538), compared with the other pT3 group (55.5%, 95% CI, 0.547–0.563; p = 0.047) and the pT4 group (46.3%; 95% CI, 0.453–0.473; p = 0.659; **Figure 2B**).

To further explore the independent factors of outcomes, univariate and multivariate analyses using Cox proportional hazards regression modeling were performed. Univariate analysis showed (**Table 2**) age at diagnosis, gender, pathological N stage, T stage, surgical modalities, and adjuvant therapy were remarkably correlated with OS and CSS (all p < 0.1), and were then incorporated into the multivariate analysis. Histology were remarkably correlated with OS in univariate analysis (p < 0.1), but were not correlated with CSS (p = 0.908). Analogously, multivariate analyses (**Table 3**) revealed that age at diagnosis, gender, pathological N stage, T stage, surgical modalities, radiotherapy and chemotherapy were independent predictive factors for both CSS and OS.

**Table 2:** Univariable analysis for overall survival (OS) and cancer-specific survival (CSS) of all patients (n=6479).

Variable	Overall survival		Cancer-specific survival	
	HR (95%CI)	*P*	HR (95%CI)	*P*
Race		0.354		0.786
white	reference		reference	
black	0.946 (0.848 - 1.056)	0.323	0.979 (0.866 - 1.107)	0.737
other	0.933 (0.828 - 1.052)	0.258	1.039 (0.912 - 1.183)	0.565
Age at diagnosis (≥ 65)	1.480 (1.387 - 1.580)	<0.001	1.338 (1.244 - 1.440)	<0.001
Gender (female)	0.814 (0.764 - 0.867)	<0.001	0.879 (0.818 - 0.944)	<0.001
Tumor location (right)	1.004 (0.943 - 1.068)	0.911	1.004 (0.935 - 1.077)	0.920
Histology		0.001		0.908
adenocarcinoma	reference		reference	
squamous-cell carcinoma	1.121 (1.050 - 1.197)	0.001	1.003 (0.930 - 1.081)	0.938
other	0.976 (0.883 - 1.080)	0.643	0.978 (0.874 - 1.094)	0.696
Pathological N stage		<0.001		<0.001
N0	reference		reference	
N1	1.276 (1.182 - 1.378)	<0.001	1.397 (1.281 - 1.523)	<0.001
N2	1.468 (1.356 - 1.588)	<0.001	1.698 (1.557 - 1.853)	<0.001
T stage (T4)	1.229 (1.154 - 1.309)	<0.001	1.345 (1.253 - 1.443)	<0.001
Surgical modalities		<0.001		<0.001
sublobectomy	reference		reference	
lobectomy or bilobectomy	0.735 (0.656 - 0.824)	<0.001	0.684 (0.604 - 0.775)	<0.001
pneumonectomy	0.892 (0.780 - 1.020)	0.095	0.856 (0.738 - 0.992)	0.039
Adjuvant therapy		<0.001		<0.001
no/unknow	reference		reference	
radiotherapy	1.291 (1.113 - 1.194)	0.001	1.395 (1.178 - 1.653)	<0.001
chemotherapy	0.677 (0.629 - 0.729)	<0.001	0.793 (0.729 - 0.862)	<0.001
chemoradiotherapy	0.932 (0.861 - 1.009)	0.084	1.098 (1.004 - 1.201)	0.041

**Table 3:** Multivariable Cox regression analysis for overall survival (OS) and cancer-specific survival (CSS) of all patients (n=6479).

Variable	Overall survival		Cancer-specific survival	
	HR (95%CI)	*P*	HR (95%CI)	*P*
Age at diagnosis (≥ 65)	1.469 (1.372 - 1.572)	<0.001	1.378 (1.278 - 1.486)	<0.001
Gender (female)	0.827 (0.775 - 0.882)	<0.001	0891 (0.829 - 0.957)	0.002
Histology		0.183		
adenocarcinoma	reference			
squamous-cell carcinoma	1.045 (0.976 - 1.118)	0.205		
other	0.955 (0.862 - 1.058)	0.377		
Pathological N stage		<0.001		<0.001
N0	reference		reference	
N1	1.401 (1.292 - 1.519)	<0.001	1.503 (1.372 - 1.646)	<0.001
N2	1.591 (1.458 - 1.736)	<0.001	1.760 (1.598 - 1.937)	<0.001
T stage (T4)	1.238 (1.161 - 1.320)	<0.001	1.323 (1.231 - 1.421)	<0.001
Surgical modalities		<0.001		<0.001
sublobectomy	reference		reference	
lobectomy or bilobectomy	0.753 (0.671 - 0.844)	<0.001	0.695 (0.613 - 0.788)	<0.001
pneumonectomy	0.852 (0.740 - 0.981)	0.025	0.773 (0.663 - 0.903)	0.001
Adjuvant therapy		<0.001		<0.001
no/unknow	reference		reference	
radiotherapy	1.190 (1.025 - 1.382)	0.023	1.252 (1.056 - 1.485)	0.01
chemotherapy	0.644 (0.596 - 0.695)	<0.001	0.729 (0.669 - 0.796)	<0.001
chemoradiotherapy	0.824 (0.754 - 0.902)	<0.001	0.908 (0.821 - 1.004)	0.06

### 3.3 The prognostic impact of rib invasion on pathological T classifications

In the comparison between the rib invasion group and the other pT3 or pT4 group, the OS and CSS rate of the rib invasion group was significantly lower than those of other pT3, but was similar to those of the pT4 group (**Figure 2A, B**).

**Figure 3:**
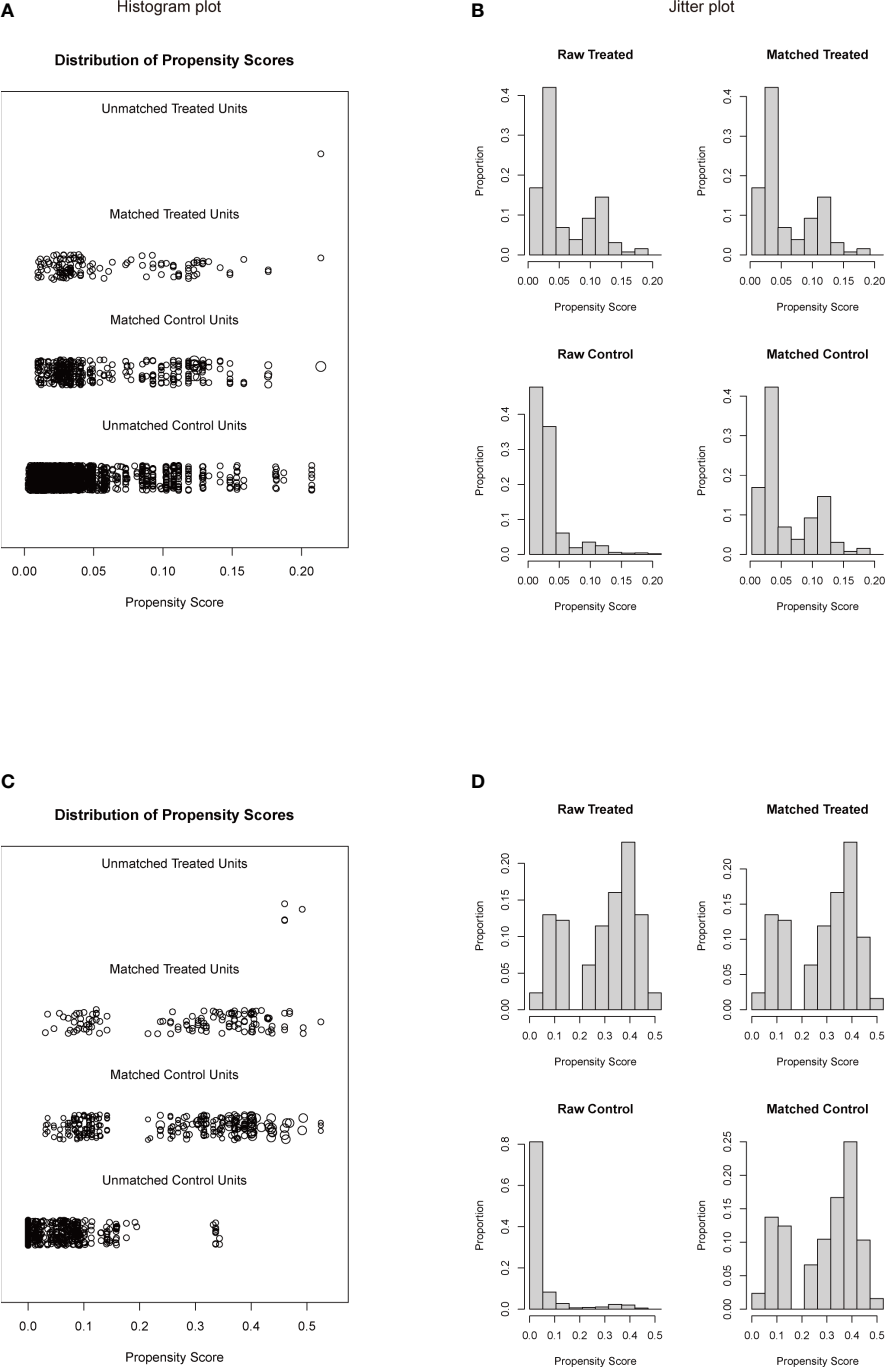
Histogram plot **(A, C)** and jitter plot **(B, D)** of PSM for rib invasion and the other pT3 or pT4 groups. **(A, B),** PSM for rib invasion and the other pT3 groups; **(C, D),** PSM for rib invasion and pT4 groups. PSM: propensity score matching. Other pT3 referred to the pT3 patients without rib invasion.

**Figure 4:**
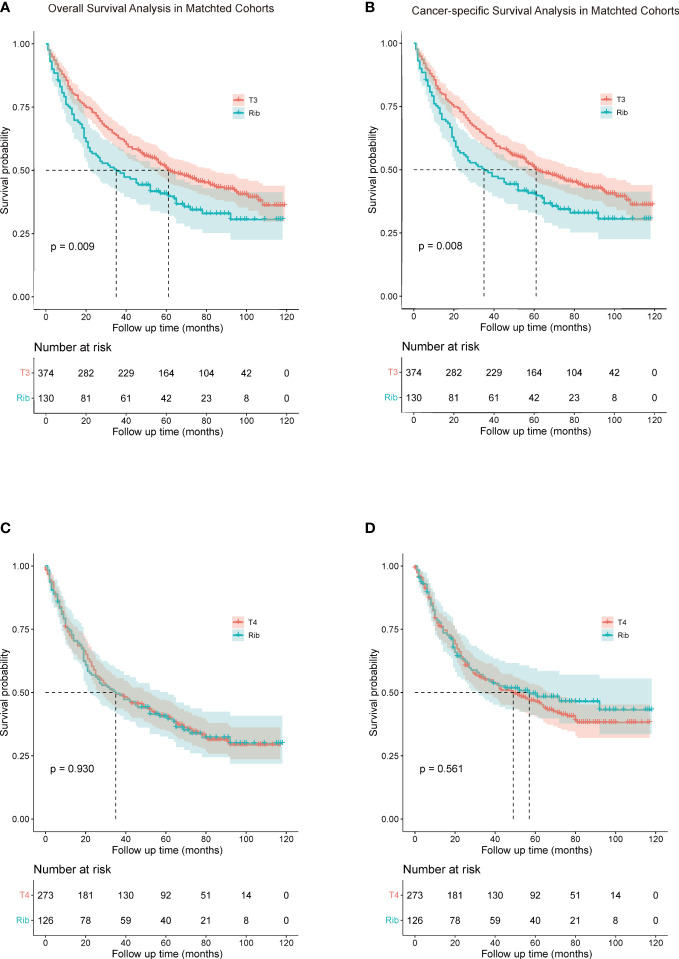
Comparison of overall survival **(A, C)** and cancer-specific survival **(B, D)** rib invasion and its matched cohort from the other pT3 and pT4 groups. **(A, B),** comparison between rib invasion and matched to the other pT3 group; **(C, D),** comparison between rib invasion and the matched pT4 group. Other pT3 referred to the pT3 patients without rib invasion.

To investigate a more appropriate pT for patients with rib invasion, 374 matched cases were generated from the other pT3 group with 130 cases from the rib invasion group; 273 matched cases from the pT4 group with 126 cases from the rib invasion group by PSM, with well-balanced baseline characteristics (**Table 4**). The histogram and jitter plot for PSM are shown in **Figure 3**. In the survival analysis for the other pT3 matched sets, the 5-year OS (40.8%, 95% CI, 0.364–0.452) and CSS rate (49.6%, 95% CI, 0.450–0.542) of rib invasion cases were both significantly lower than those of the other pT3 cases (OS: 50.9%, 95% CI, 0.483–0.535; p = 0.009; CSS: 60.0%, 95% CI, 0.573–0.627; p = 0.008; **Figure 4A, B**). In the matched pT4 sets, the rib invasion had a similar 5- year OS rate (40.5%, 95% CI, 0.361–0.449), and 5-year CSS rate (49.5%, 95% CI, 0.448–0.542) with matched pT4 cases (OS: 40.4%, 95% CI, 0.374–0.434; p = 0.930; CSS: 46.3%, 95% CI, 0.431–0.495; p = 0.561; **Figure 4C, D**).

**Table 4:** Baseline characteristics for patients with rib invasion in the matched cohorts.

Characteristic	Rib (n=130)	T3 (n=374)	*P*	Rib (n=126)	T4 (n=273)	*P*
Age at diagnosis, years			<0.001			<0.001
< 65	54 (41.54)	149 (39.84)		53 (42.06)	115 (42.12)	
≥ 65	76 (58.46)	225 (60.16)		73 (57.94)	158 (57.88)	
Race, n (%)			0.403			0.824
white	112 (86.15)	343 (91.71)		107 (84.92)	234 (85.71)	
black	14 (10.77)	22 (5.88)		15 (11.91)	32 (11.72)	
other	4 (3.08)	9 (2.41)		4 (3.17)	7 (2.57)	
Gender, n (%)			0.001			0.029
male	79 (60.77)	229 (61.23)		75 (59.52)	159 (58.24)	
female	51 (39.23)	145 (38.77)		51 (40.48)	114 (41.76)	
Tumor location, n (%)			0.367			0.781
left	52 (40.00)	151 (40.37)		52 (41.27)	129 (47.25)	
right	78 (60.00)	223 (59.63)		74 (58.73)	144 (52.75)	
Histology, n (%)			0.165			0.310
adenocarcinoma	50 (38.46)	141 (37.70)		50 (39.68)	109 (39.93)	
squamous-cell carcinoma	56 (43.08)	164 (43.85)		51 (40.48)	119 (43.59)	
other	24 (18.46)	69 (18.45)		25 (19.84)	45 (16.48)	
Pathological N stage, n (%)			0.015			0.081
N0	107 (82.31)	308 (82.35)		103 (81.75)	203 (74.36)	
N1	14 (10.77)	38 (10.16)		14 (11.11)	48 (17.58)	
N2	9 (6.92)	28 (7.49)		9 (7.14)	22 (8.06)	
Pathological size (cm)			0.715			0.635
≤ 3	12 (9.23)	34 (9.09)		12 (9.52)	38 (13.92)	
> 3cm, ≤ 5cm	52 (40.00)	138 (36.90)		53 (42.06)	125 (45.79)	
> 5cm, ≤ 7cm	66 (50.77)	202 (54.01)		61 (48.41)	110 (40.29)	
> 7	0	0		0	0	
Surgical modalities, n (%)			0.005			0.019
sublobe	12 (9.23)	38 (10.16)		12 (9.52)	44 (16.12)	
lobectomy or bilobectomy	115 (88.46)	305 (81.55)		111 (88.10)	161 (58.97)	
pneumonectomy	3 (2.31)	31 (8.29)		3 (2.38)	68 (24.91)	
Adjuvant therapy, n (%)			0.35			0.617
no/unknow	67 (51.54)	64 (17.11)		63 (50.00)	105 (38.46)	
radiotherapy	26 (20.00)	99 (26.47)		27 (21.43)	75 (27.47)	
chemotherapy	9 (6.92)	18 (4.81)		9 (7.14)	16 (5.86)	
chemoradiotherapy	28 (21.54)	193 (51.61)		27 (21.43)	77 (28.21)	

Rib, rib invasion group; T3, the other pT3 group (the pT3 patients without rib invasion); T4, pT4 group.

## 4 Discussion

The prognostic significance of the degree of the chest wall invasion was not reflected in the 7^th^ TNM classification. Instead, the chest wall invasion was classified into the T3 stage for NSCLC patients in the 8^th^ TNM system. However, pT3 lung cancer was described directly according to the depth of chest wall invasion as follows: pT3a, invasion up to PL3; pT3b, invasion to the endothoracic fascia; and pT3c, invasion up to the ribs or soft tissues in the General Rule for Clinical and Pathological Record of Lung Cancer in Japan (16). An extensive review of the literature reveals that most of the articles only studied pT3 NSCLC concerning chest wall involvement only (3, 5, 10, 12, 17). There was only one study that further evaluated the impact of rib invasion on the pathological T classifications for NSCLC until now (14). This study analyzed the effect of rib invasion for NSCLC patients using the SEER database, and provids a more appropriate pT classification for consideration in the forthcoming 9^th^ TNM classifications.

In the past, many researchers have explored the impact of chest wall invasion on the survival of patients with NSCLC, but there is still no consensus. Marc Riquet et al. (4) analyzed the characteristics and prognosis of resected T3 NSCLC, indicating that chest wall pT3 subgroup 5-year survival was neither significantly modified by tumor size nor by N involvement nor in-depth chest wall invasion despite a poor survival in cases with N1 to 2 diseases and pleura invasion. Harold M. Burkhart et al. (7) included 94 patients who underwent 95 en bloc lung and chest wall resections. The survival analysis revealed that the 5-year survival for tumors that invaded the parietal pleura was only 49.9%, that for tumors that invaded soft tissue and the parietal pleura was 35.0%, and that for tumors that invaded bone was 31.6%, respectively. These differences were not statistically significant. They also revealed that all the earlier reports (3, 18, 19) from their institution did not demonstrate the depth of chest wall invasion as a predictor of survival. Recently, Marco Chiappetta et al. (8) examined the association between chest wall invasion, resection type, nodal status, and survival. Comparing only pleural invasion with soft tissue/rib invasion, no 5-year survival difference was found either in all stages (25 *vs.* 28%, respectively, p = 0.78) or in IIB (T3N0) stage (29 *vs.* 57%, respectively, p = 0.68). They found that no correlation between survival and kind of resection or extension of tumor invasion in the soft tissues or bones was found. However, in 2006, Luca Voltolini et al. (10) found a statistical difference in 5-year survival between patients with a tumor infiltration confined to parietal pleura and those with infiltration of the chest wall (p = 0.003). Alain Chapelier et al. (20) investigated possible factors which could affect long-term survival following radical resection of lung cancer with chest wall invasion. They found that the extension of resected chest wall, nodal status, and mainly the histological differentiation and depth of chest wall involvement were factors affecting long-term survival after complete resection. Deeper involvement was advocated as a predictor of worse prognosis in a recent report by Facciolo et al. (17). Our study showed significantly lower OS in patients with rib invasion than in those with other pT3 (40.5% *vs*. 46.5%, p = 0.035). Similar results were also obtained in patients after PSM. The result was similar to Zhao et al. (14) who found that the depth of chest wall invasion was an independent prognostic factor for NSCLC, and the 5-year survival rate of the rib invasion group was significantly lower than that of more superficial invasion groups. Our study included a large sample from multicenter patients in the United States. Our results also showed that favorable prognostic factors included age at diagnosis (< 65), gender (female), surgical modalities (lobectomy, bilobectomy or pneumonectomy), no radiotherapy and chemotherapy.

In terms of gender, we revealed that women have a better prognosis than men in NSCLC patients with T3 and T4 disease. Many studies showed that women have a higher survival rate than men among NSCLC patients. De Perrot et al. (21) found that there were striking differences in smoking habits between men and women, which resulted in different clinical presentation, histology, treatment and prognosis. Hiroyuki Sakurai et al. (22) concluded that women had a much higher incidence of adenocarcinoma than men. In addition, women with adenocarcinoma had a significantly better prognosis in both stage I disease and stage II disease. Katsuya Watanabe et al. (23) revealed that the hazard rate and the peak times of recurrence after resection of NSCLC differed considerably between men and women. Women having a longer disease-free interval within subsets of the same disease stage, histological type, and smoking status, which might account for the better survival.

Whether adjuvant therapy could improve the prognosis of NSCLC patients with chest wall invasion remains unclear. According to the multivariate analyses, we point out that chemotherapy could improve the survival outcomes of NSCLC patients with T3 and T4 disease, while radiotherapy and chemoradiotherapy not, which is different from the results of the following studies. Gould et al. (24) concluded that although adjuvant radiotherapy may reduce local recurrence, it has no impact on long-term survival. Luca Voltolini et al. (10) revealed that there were no differences in survival observed between patients who underwent adjuvant treatment and those who did not. They concluded that preoperative radiation therapy could improve the success of resection by circumscribing and reducing the infiltration of the chest wall, thus, achieving a final condition closer to isolated involvement of the parietal pleura. Lisa M. Brown et al. (25) showed no survival benefit with adjuvant chemotherapy for pT3N0M0 NSCLC with chest wall invasion, regardless of tumor size or grade, after R0 resection. However, Chiappetta et al. (8) included 59 patients who underwent surgery with a curative intent for NSCLC invading chest wall structures, and found that only radiotherapy affected survival in some subgroups of patients, with a 2-year survival rate of 100% in N1 to N2 patients who underwent radiotherapy compared to 0% in those untreated (p = 0.07). Therefore, whether adjuvant therapy improves long-term survival in NSCLC patients with chest wall invasion remains controversial and requires further evaluation.

We hold the opinion that rib invasion should be reclassified into pT4 disease due to the similar risk of dying. Should rib invasion be treated as a contraindication to surgery if modified pT for rib invasion is adopted? In our study, surgical modalities was an independent risk factor for both CSS and OS, however, the surgical modalities to the chest wall lesions was not completely recorded. Several studies have analyzed the surgical outcomes of NSCLC with chest wall invasion. In the past, only surgical treatment was considered effective for patients with chest wall invasion, and there was no consensus on a specific treatment. Many studies (12, 17, 26, 27) have shown that there is no significant difference in the long-term survival between extrapleural resection and en bloc chest wall resection for NSCLC patients with different depths of chest wall invasion. However, R0 resections improved the survival of patients compared with R1 and R2 resections. In early 1999, Robert J. Downey et al. (26) showed that survival after resection of NSCLC involving the chest wall is significantly related to the completeness of resection and the presence of nodal metastases. Chang Young Lee et al. (12) revealed that complete resection (R0) and the absence of N2 disease were revealed as better prognostic factors for long-term survival of patients with chest wall invading NSCLC. As previously reported (9, 12, 17, 26), lymph node status is highly correlated with long-term survival, and the survival rate decreases significantly with an increase of the degree of lymph node involvement. Therefore, preoperative mediastinoscopy should be used to assess the lymph node status of NSCLC patients suspected of severe lymph node involvement. We suggest that preoperative evaluation, such as chest CT, magnetic resonance imaging (MRI), bone scintigraphy, ultrasound (US) examination, and Fluorine 18 fluorodeoxyglucose (FDG) positron emission tomography (PET-CT), should be performed in suspected chest wall invasion NSCLC patients. Complete resection is the key point of surgery for chest wall invasion patients with N0 stage, while for patients with positive lymph nodes, neoadjuvant chemotherapy can be considered.

This study has several limitations. Firstly, our study was retrospective, and had a relatively homogeneous cohort, and selection bias was unavoidable. Secondly, the study included multicenters patients in the USA and few details were available about different surgeon skill levels and surgical techniques which might affect the outcomes. Thirdly, several covariates such as age at diagnosis and gender could not be well balanced, even after PSM. Finally, the records of surgical modalities and chemotherapy were insufficiently detailed in the SEER database, and we were unable to evaluate the effects of surgical modalities and chemotherapy regimens on the prognosis. In recent years, along with the development of immunotherapy, many patients with advanced NSCLC have regained access to surgery after immune-combined chemotherapy or targeted therapy, and the survival rate has been significantly improved. It is worthwhile to further investigate whether preoperative immune-combination chemotherapy or targeted therapy should be given to patients with chest wall infiltrating NSCLC.

In conclusion, we found that the survival outcome of the rib invasion group was worse than the other pT3 group, but similar to the pT4 group. The suitability of rib invasion for upstaging to pT4 should be further validated in the forthcoming 9^th^ TNM classification. The revision of rib invasion in the staging system will help to further determine clinical treatment.

## Data availability statement

Publicly available datasets were analyzed in this study. This data can be found here: https://seer.cancer.gov/data/access.html.

## Ethics statement

Ethical review and approval was not required for the study on human participants in accordance with the local legislation and institutional requirements. Written informed consent for participation was not required for this study in accordance with the national legislation and the institutional requirements.

## Author contributions

YC: Conceptualization; Methodology; Writing-original draft; Writing-review and editing. JZ: Data curation; Formal analysis; Writing-review and editing. JC: Formal analysis; Writing-review and editing. ZY: Data curation; Software; Formal analysis. YD: Data curation. WC: Writing-review and editing. TG: Methodology; Resources; Supervision. LZ: Supervision; Writing-review and editing. XP: Project administration; Supervision; Writing-review and editing. All authors contributed to the article and approved the submitted version.
